# Measuring social integration and tie strength with smartphone and survey data

**DOI:** 10.1371/journal.pone.0200678

**Published:** 2018-08-23

**Authors:** Agnete S. Dissing, Cynthia M. Lakon, Thomas A. Gerds, Naja H. Rod, Rikke Lund

**Affiliations:** 1 Section of Social Medicine, Department of Public Health, University of Copenhagen, Copenhagen K, Denmark; 2 Program in Public Health, University of California Irvine, Irvine, CA, United States of America; 3 Section of Biostatistics, Department of Public Health, University of Copenhagen, Copenhagen K, Denmark; 4 Section of Epidemiology, Department of Public Health, University of Copenhagen, Copenhagen K, Denmark; 5 Copenhagen Stress Research Centre, Copenhagen, Denmark; 6 Center for Healthy Aging, Faculty of Health Sciences, University of Copenhagen, Copenhagen N, Denmark; University of Rijeka, CROATIA

## Abstract

Recordings of smartphone use for contacts are increasingly being used as alternative or supplementary measurement methods for social interactions and social relations in the health sciences. Less work has been done to understand how these measures compare with widely used survey-based information. Using data from the Copenhagen Network Study, we investigated whether derived survey and smartphone measures on two widely studied concepts; Social integration and Tie strength were associated. The study population included 737 college students (mean age 21.6 years, Standard deviation: 2.6), who were followed with surveys and continuous recordings of smartphone usage over a one-month period. We derived self-reported and smartphone measures of social integration (social role diversity, social network size), and tie strength (contact frequency, duration and tie reciprocity). Logistic regression models were used to assess the associations between smartphone derived and self-reported measures adjusting for gender, age and co-habitation. Larger call and text message networks were associated with having a high self-reported social role diversity, and a high self-reported social contact frequency was likewise associated with having both frequent call and text message interactions, longer call duration and a higher level of reciprocity in call and text message communication. Self-reported aspects of social relations and smartphone measures of social interactions have considerable overlap supporting a measurement of similar underlying concepts.

## Introduction

Social relations are important to human health. Both structural aspects such as network size and contact frequency as well as functional aspects such as social support has been established as important determinants of human health and well-being during the last decades [1–5]. Most of this evidence is based on self-reports from surveys, however, alternative ways of measuring social relations are emerging. Over the last decade, smartphones have become increasingly available and they provide a previously unthinkable framework for gaining detailed insights into human social interaction. Phone calls, online comments, GPS location and Wi-Fi-login are automatically recorded [6, 7]. These kinds of ‘big data’ provide fine grained information on human social interactions over time and place [7–12], and are increasingly being used to study social relationships in relation to health [8, 11–15], but also in relation to other areas such as transportation [16], political opinions [17], economic opportunities [18], and information spread [19]. Despite the increasing use of these data, less work has been carried out to evaluate how smartphone measures of social relations compare with more widely-used survey-based methods. Investigating whether survey and smartphone measures capture underlying theoretical concepts is important in order to further advance the use of smartphone measures of social relations.

Smartphone data reflect human social interaction by objectively recording social events happening between individuals at one point in time via different communication channels such as calls, texts, and proximity recordings by Bluetooth scans, and hereby also record interactions that individuals do not remember or perceive as being important [20]. Self-reports of social relations often reflect individual evaluations of social ties that exist over a longer time span [21]. Nevertheless, despite the distinct differences inherent in the two types of data, it is possible that there is an overlap as both methods reflect social connectivity, and using both might be complementary for measuring individual level social relationships [20, 22]. In order to use smartphones as measurement tools to study individual social relations, we propose to derive relevant smartphone measures of social relations based on theoretical concepts, and to further explore the content of such smartphone measures.

### Social integration

**Fig 1:**
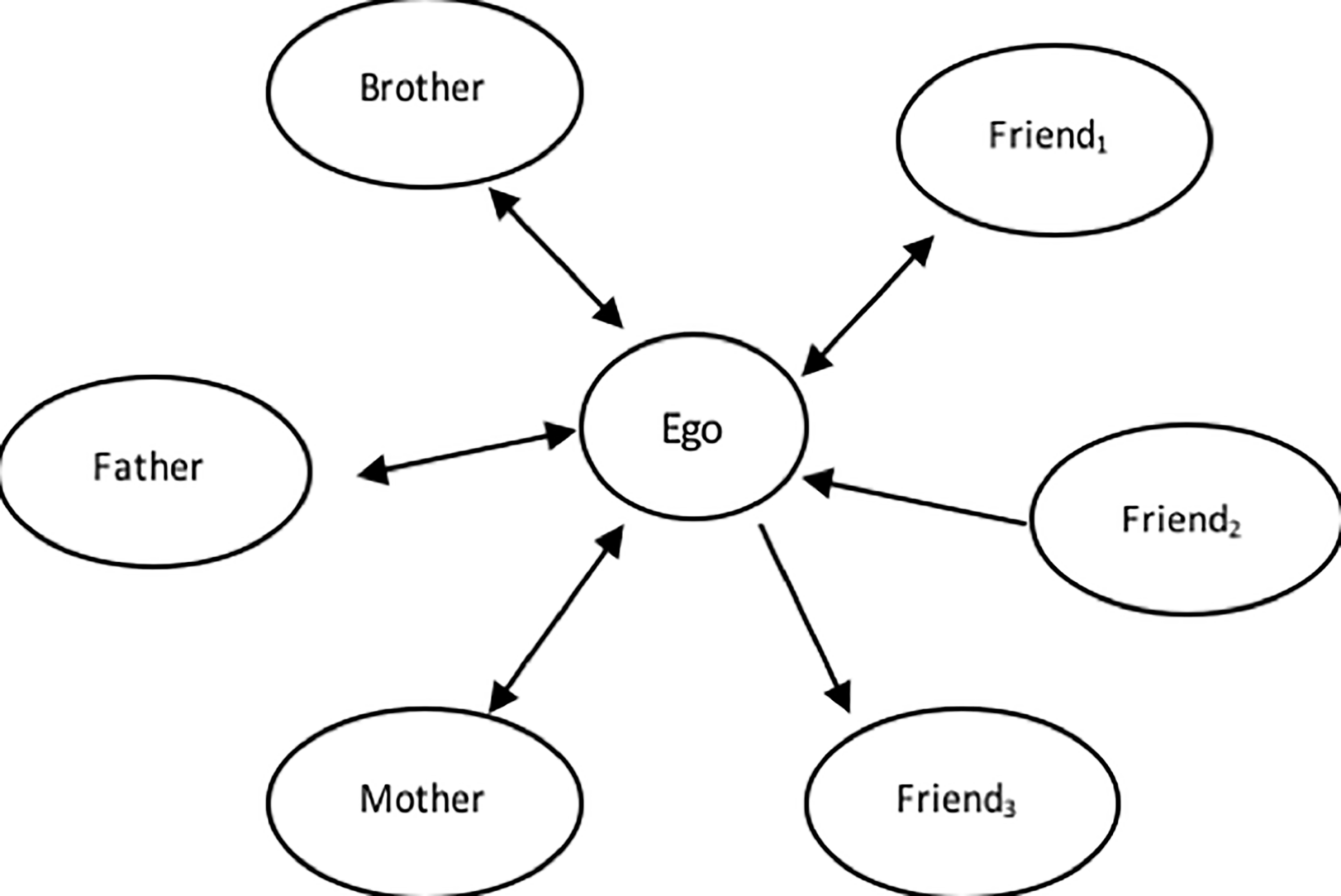
Illustration of social network size, social role diversity, social ties (tie strength, and reciprocity). Fig 1 illustrates definitions of social relation concepts. *The Diversity* is defined as the number of different social roles (e.g. friend, brother, mother), *The Network Size* is defined as the number of social ties (all arrows in the figure), *The Tie Strength* is defined as a continuum of closeness in a relationship ranging from weak ties to strong ties (Thickness of arrow). *The Reciprocity* is defined as the mutuality in a social tie and describes a social tie between two individuals where the tie is directed both ways (bi-directional arrow).

Social integration has been widely studied in relation to well-being and health outcomes, and the literature is rich in various ways of defining and measuring this construct [23–27]. This multifaceted construct has been conceptualized as the extent to which individuals are connected within and participate in a broad range of social relations and activities reflecting how well the individual is connected within a social network [25–27]. Two concepts can be used to operationalize social integration: the *social network size* and the *social role diversity* [26]. The Social network size is defined as the number of social ties connected to an individual, and hence reflect how well an individual is connected within a social network. In contrast to counting the sheer number of social ties, social role diversity indicates the number of different social roles surrounding the individual. A person might engage in social interaction with a friend in one social context, a sibling in a second, and with a parent in yet another context. Fig 1 depicts social network size and the social role diversity for one individual (ego). The figure shows a network size of seven, but a social role diversity of five as some of the social ties are characterized by having the same social role, e.g. friendship. Nonetheless, both measures reflect the extent to which an individual participates in different contexts involving various social activities, and hereby also provide insight into information about one’s level of social integration. Existing survey instruments evaluating diversity include social roles relating to both family, non-family roles such as friends and neighbours and roles from local community activities [1, 27, 28]. Given that having a high social role diversity relates to being in contact with a wide range of different people [26], a high social role diversity might also be reflected in social interaction behavior via smartphones, with social integration in this context meaning a larger communication network. Nevertheless, this hypothesis is largely empirically untested. We identified one study investigating network size of call patterns, but this smartphone measure was not directly compared against a self-reported measure [29].

### Tie strength

Social ties can be characterized by their strength. Tie strength is a continuum of closeness in a relationship, ranging from weak ties (narrow arrows in Fig 1), to strong ties which are ties to people with whom the individual has an intimate relationship with, for example, a close friend or a family member (bold arrows in Fig 1). Tie strength has been defined as “..a combination of the amount of time, the emotional intensity, the intimacy (mutual confiding), and reciprocal services that characterizes the tie” [30], and hence used indicators of tie strength include contact frequency, tie duration, and tie reciprocity [31]. The *Contact frequency* is the sum of social interactions in a defined time period, which can be carried out in a face-to-face encounter or in a mediated encounter, e.g. social contact via a smartphone. The *Duration* considers the duration of social interactions, where longer duration is an indicator of strong ties [30, 32]. *Tie reciprocity* describes the mutuality in a social tie (illustrated by the bi-directional arrow in Fig 1), where mutual relationships are indicators of strong ties [30]. These tie strength indicators are considered closely related as they measure the same underlying concept of tie strength [33]. The indicators can be described as interactional network characteristics as they reflect the extent of interactions a respondent has with alters, i.e. surrounding individuals. As such these indicators describe the structure in which respondent’s perception of closeness in a social tie is likely to occur. In the relatively sparse literature comparing smartphone data to survey data, predicting self-reported closeness in a social tie with smartphone data has most often been done [20, 22, 29, 34]. Studies using measures such as frequency of calls [34], reciprocity in call patterns [35], as well as face-to-face proximity recorded with Bluetooth data [20, 22] have been able to predict self-reported closeness in a social tie. As smartphone data represent count data, they might be superior in evaluating sheer structural aspects of tie strength for example contact frequency. In the paper, we will emphasize the structural aspects of tie strength as we consider smartphone data adequate for measuring such aspects.

In this paper, we conduct conceptual work aiming to understand aspects of social integration, and tie strength captured in smartphone data. We hypothesize that derived information on social interactions collected continuously via smartphones during a one-month period is associated with self-reported measures of social integration and tie strength. We do not attempt to validate smartphone measures against a golden survey standard of social relations measurement as such does not exist.

## Material and methods

### Study population

We used data from the Copenhagen Network Study, which was established to study social activity and behaviors based on smartphone data among young adults [36]. In September 2013, 3,329 undergraduate students at a large technical university in Denmark were invited to participate in the study of which 979 students accepted the invitation (response rate = 29%). The majority of the participating students were freshmen students (60%). All participants received a smartphone (LG NEXUS 4) in which they inserted their private SIM-card to make it their primary communication device. The smartphone was running customized software recording all outgoing and ingoing call and text messages with related timestamps as well as unique identifiers for each contacted alter. Before receiving the smartphone, the students completed a baseline questionnaire on self-reported social relations. A detailed description of the high-resolution smartphone data collection can be seen in Stopczynski et al. 2014 [36]. The participants were recruited continuously throughout the year, and the used smartphone data were recorded continuously in one month from receiving the smartphone and responding to the baseline questionnaire. We excluded individuals with no information on self-reported social relations (N = 33), and with missing smartphone recordings (N = 209) yielding a total sample of 737 participants. Missing smartphone data was not related to gender (chi-squared test, p-value = 0.80), but was related to age where younger students were more likely to have their smartphone data recorded (t test, p-value = 0.026).

### Measurements

To measure the concepts of social integration and tie strength, we used validated survey items from the Copenhagen Social Relations Questionnaire [37], and derived smartphone data from both call and text messages. Table 1 shows a summary of the measures used.

**Table 1:** Overview of concepts, operationalization, data types and measures.

Concept	Operationalization	Data type	Measure
**Social integration**	Social role diversity	Survey data	Number of social roles with frequent contact (contact one to three times per month or more) both face-to-face and non-face-to-face with roles; Mother, Father, Friends, Partner, Siblings, Extended family)
	Social network size	Smartphone data	Number of alters called and texted at least once during a month
**Tie strength**	Total contact frequency	Survey data	Total face-to-face and non-face-to-face contact frequency with all roles (Mother, Father, Friends, Partner, Siblings, Extended family)
	Contact frequency, Tie duration, Tie reciprocity	Smartphone data	Frequency of calls and texts per alter per month, Total duration of calls per month, number of alters with reciprocated activity (both placing and receiving at least one call or text from the same alter).

#### Social integration

As a measure of the *Social role diversity*, we assessed the number of six different social roles for which the participant self-reported frequent face-to-face contact using the item: How often are you together with any of the following people (mother, father, siblings, extended family, partner, and friends), who you do not live with? (Response code: Several days a week; About once a week; One to three times a month; Less often than once a month; Never; Have no; Live with). The family and non-family social roles were considered relevant for a population of college students who are in a transitional stage of life increasingly creating close relations with peers, but still relying on close family members for support, mutual confiding and information [38]. A contact frequency of one to three times a month or more as well as co-habitation was considered frequent contact corresponding to active social role participation defined elsewhere [26]. Having less frequent social contact or having no social role was used as an indicator that no role was present. The derived variable of the number of social roles was categorized in intervals of two. One participant had no social roles and was grouped in the lowest category. Similarly, we also assessed the social role diversity in non-face-to-face contacts for the same six social roles using the item: How often do you have contact with any of the following people without seeing them (e.g. by telephone, letters, e-mail, SMS)? From the smartphone data, we derived a measure of the *Social network size* by counting the number of different alters that the participant had interacted with at least once in a month via either placed *or* received calls and text messages. The social network size values were grouped in intervals of ten.

#### Tie strength

From the self-reported face-to-face and non-face-to-face contact frequency survey items described above, we derived a composite measure indicating the *Total contact frequency* with all social roles. We summed the six contact frequency items containing five categories (coded 0–4) on a scale from 0–24 where a score of 24 indicated reporting “Several days a week” or “Live with” for all social roles and a score of 0 indicated reporting “Never” or “Have no” for all roles. In order to maintain important information on strong social ties, individuals reporting “live with” was grouped in the highest contact frequency category. The summary scale was grouped in five-interval categories. We developed a similar measure for the total non-face-to-face contact frequency. From the smartphone data, we also derived a measure of total contact frequency by summing of the total number of calls and text messages during a month normalized by the number of alters in the social network. The call interaction variable was grouped in intervals of three, and the text interaction variable was grouped in intervals of fifteen. Further, to construct an indicator of the *total duration* of social interactions, we summed the duration of calls for each individual during a month and grouped this variable in one-hour intervals. Non-received calls were excluded. As a measure of *tie reciprocity*, we counted for each respondent the number of ties with reciprocated smartphone activity defined as having both placed *and* received at least one call or text message from the same alter. These variables were grouped in three intervals for call reciprocity and six intervals for text message reciprocity. Nine participants did not have call activity and hence were excluded from this variable as reciprocation in this situation was not meaningful. We did not have survey data available indicating duration and reciprocity in social ties.

### Analytical strategy

We investigated distributions of age, gender, self-reported and derived smartphone measures of social integration and tie strength in the study population. To assess the association between the self-reported and smartphone variables of social integration, we estimated odds ratios and 95% confidence intervals using logistic regression models with the highest social role diversity category as outcome category (5–6 social roles). Likewise, associations between smartphone measures of contact frequency, duration and reciprocity and self-reported measures of total contact frequency were evaluated using the highest self-reported total contact frequency as outcome, i.e. scoring between 20–24 on the derived total contact frequency summary scale. The evidence of trends in associations, e.g. whether the odds of having high social role diversity was increasing with a larger call network, was assessed by including the categorized smartphone measure as continuous in the logistic regression model. As social relations and smartphone usage vary with age and gender [39, 40], and as calling behavior of young adults appears to be influenced by whether they live with their parents [38], all models were adjusted for age, gender and co-habitation (living with at least one of the six social roles). We conducted the following sensitivity analyses: 1) Counting co-habitation as an active social role might categorize some individuals as having a high social integration despite having few friends, e.g. individuals living at home with family members but indicating infrequent contact with friends. Hence, we conducted a sensitivity analysis excluding co-habiting individuals to assess whether the results were robust to this categorization. 2) To assess whether the results were robust to dichotomizing self-reported social relation variables in high versus low for use in logistic regression models, we also conducted linear regression analyses using a continuous version of the measures. 3) As the smartphone measure of social network size was in risk of being wrongly categorized due to service call and alike, we restricted the measure to a definition where network size was counted as the number of unique persons called/texted at least three times in a sensitivity analysis. All analyses were conducted using statistical software R version 3.3.3. [41].

### Ethics statement

All participants gave informed consent and were able to withdraw from the study at any time. All data were used anonymously, and The Copenhagen Network Study was approved by the Danish Data Protection Agency (approval number: 2012-41-0664).

## Results

### Descriptive statistics

The majority of the population were males (77%), and the mean age was 21.6 year (SD: 2.6) ranging from 18 to 46 years. Distributions of smartphone measures can be seen in S1 Table. The majority of participants were in call (38.8%) and text (40.4%) contact at least once with between 11 and 20 different alters in a month. Further, the majority of the study population had between 4–6 call interactions (51.6%) and 0–14 text interactions (40.4%) per alter in a month. The majority of the study population had a call duration of 3 hours or more during a month (38.9%), and between 4–6 reciprocated call (33.7%) and more than 18 reciprocated text message ties (32.7%). Women had larger text message networks, a higher text message frequency, longer call duration and a higher text message reciprocity compared to men. The mean age was higher among participants with a large call network and a long call duration, but lower for participants with a high text message frequency. Distributions of self-reported survey measures can be seen in S2 Table. 41.7% and 56.9% of the study population had a high social role diversity in face-to-face contact and non-face-to-face contact, respectively. 6.1% and 15.5% scored between 20–24 on the total face-to-face and non-face-to-face contact frequency summary scale, respectively. 315 individuals reported to be co-habiting with a social role where the majority of these (46%) were living with their parents. Participants with a higher social role diversity and self-reported contact frequency appeared to be younger than participants with lower contact frequency and role diversity.

### Social integration

**Fig 2:**
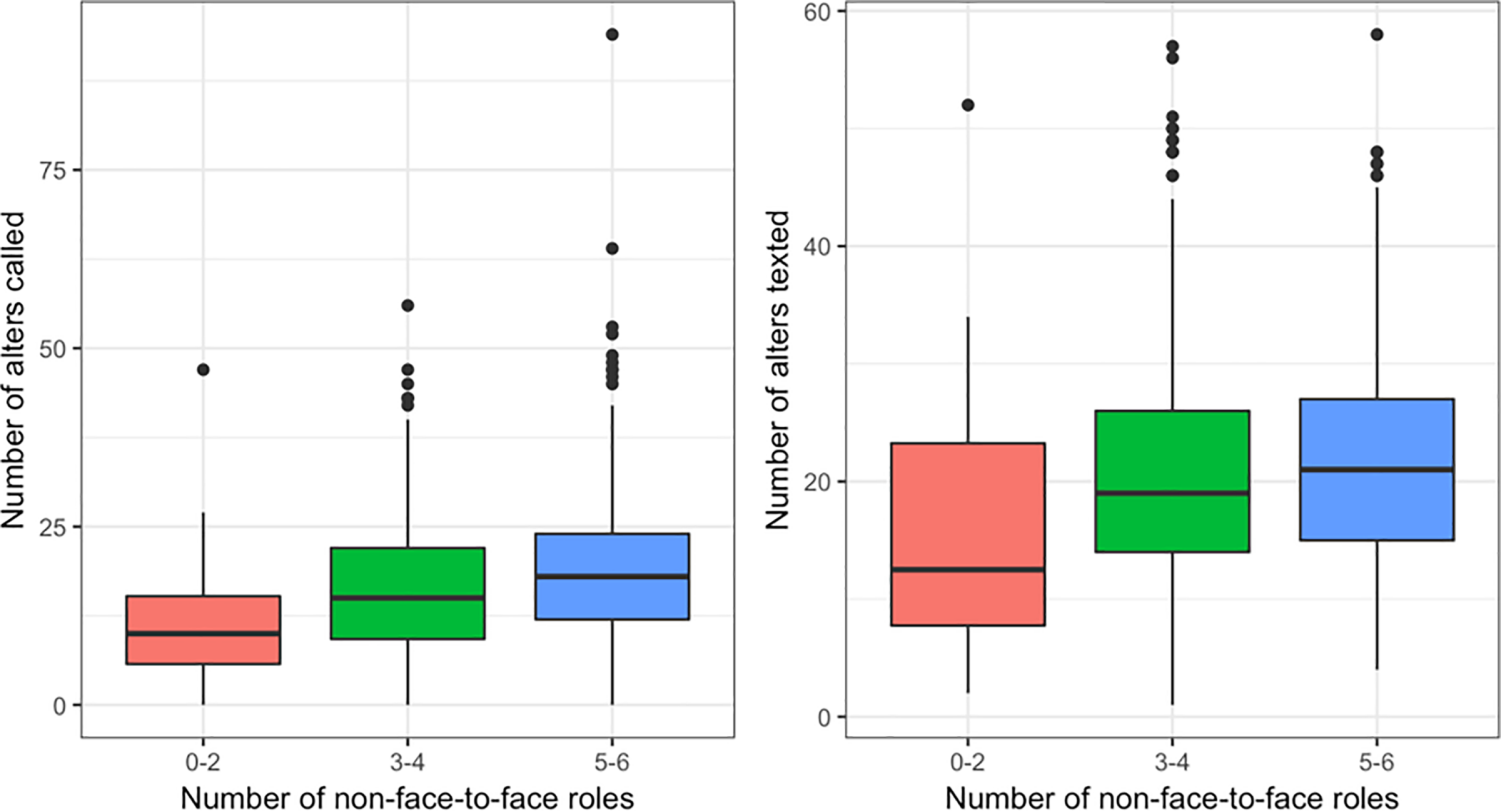
Boxplots of the associations between self-reported social role diversity and smartphone derived measures of social network size. Fig 2 illustrates medians (midline of box), 25^th^ and 75^th^ percentiles (upper and lower edge of box) of smartphone measured social network size in groups of self-reported non-face-to-face social role diversity.

Fig 2 shows medians of smartphone network size in groups of self-reported social role diversity. From the figure it is apparent, as hypothesized, that the median of alters contacted via smartphones is increasing with the number of self-reported social roles. This tendency is confirmed when adjusting for age, gender and co-habitation (Table 2). Participants having contact with more than 30 different alters via calls and text messages have more than two times higher odds (Call: OR = 2.58, 95%CI:1.50;4.44) (Text: OR = 2.55, 95%CI: 1.44;4.50) of also having frequent non-face-to-face contact with 5–6 social roles compared to participants who have call and text message contact with only 0–10 alters. The same pattern appears for self-reported face-to-face diversity (Table 2). There is strong evidence for trends in the reported associations suggesting that the odds ratios for having a high social role diversity is increasing with a larger call and text message network. Using a continuous version of the self-reported social relation variable did not change the overall conclusion of these findings (S3 Table). Further, excluding co-habiting individuals as well as using a more restricted definition of call and text message network size did not change the conclusion of the results although the association appeared to be more pronounced for non-face-to-face diversity (S4 and S5 Tables).

**Table 2:** Associations between smartphone and self-reported measures of social integration in a population of 737 young adults.

	Total population	High social role diversity (Frequent face-to-face contact with 5–6 social roles)		High social role diversity (Frequent non-face-to-face contact with 5–6 social roles)	
Smartphone social network size per month	N (%)	OR	95%CI	OR	95%CI
*Number of alters called *					
0–10 alters	186 (25.2)	1	(Ref)	1	(Ref)
11–20 alters	286 (38.8)	1.26	(0.85;1.87)	1.91	(1.30;2.79)
21–30 alters	181 (24.6)	1.50	(0.97;2.32)	2.78	(1.80;4.28)
More than 30 alters	84 (11.4)	1.82	(1.05;3.14)	2.58	(1.50;4.44)
P-value (test for trend)		0.018		<0.0001	
*Number of alters texted*	* *				
0–10 alters	92 (12.5)	1	[Ref]	1	(Ref)
11–20 alters	298 (40.4)	1.99	(1.18;3.34)	1.64	(1.02;2.64)
21–30 alters	223 (30.3)	2.26	(1.31;3.88)	2.20	(1.33;3.63)
More than 30 alters	124 (16.8)	2.31	(1.26;4.22)	2.55	(1.44;4.50)
P-value (test for trend)		0.014		0.0005	

OR = Odds ratio, 95%CI = 95% confidence interval. All OR adjusted for age, gender and co-habitation.

### Tie strength

A higher smartphone contact frequency of calls and text messages is also strongly associated with self-reported face-to-face contact frequency. Having more than 9 calls per alter is associated with six times higher odds (OR = 6.53, 95%CI:1.98;21.47) of also scoring high on the self-reported contact frequency scale (20–24) compared to having between 0–3 calls per alter. The same tendency appeared for the self-reported non-face-to-face contact frequency, which also showed strong associations with call interaction frequency. Longer call duration and higher reciprocity in call and text message communication was associated with reporting a high face-to-face contact frequency, and longer call duration and higher reciprocity in call communication was associated with reporting a high non-face-to-face contact frequency (Table 3). Using a continuous version of the measures did not change these conclusions although there was no association between call duration and face-to-face contact frequency (S3 Table).

**Table 3:** Associations between smartphone and self-reported measures of tie strength in a population of 737 young adults.

	Total population	High face-to-face contact frequency (summary score = 20–24)		High non-face-to-face contact frequency (summary score = 20–24)	
Smartphone measures per month	N (%)	OR	95%CI	OR	95%CI
*Frequency of call interactions per alter*	* *				
0–3 calls	221 (30.0)	1	(Ref)	1	(Ref)
4–6 calls	380 (51.6)	2.85	(1.13;7.16)	2.36	(1.34;4.16)
7–9 calls	96 (13.0)	2.51	(0.77;8.22)	3.10	(1.54;6.28)
More than 9 calls	40 (5.4)	6.53	(1.98;21.47)	5.82	(2.55;13.28)
P-value (test for trend)		0.004		<0.0001	
*Frequency of text interactions per alter*	* *				
0–14 texts	298 (40.4)	1	(Ref)	1	(Ref)
15–29 texts	240 (32.6)	5.92	(1.94;18.10)	1.80	(1.09;2.98)
30–44 texts	100 (13.6)	5.48	(1.57;19.15)	2.01	(1.08;3.74)
More than 45 texts	99 (13.4)	11.44	(3.63;36.04)	1.58	(0.83;3.02)
P-value (test for trend)		<0.0001		0.072	
*Call duration*					
up to 1 hr	160 (21.7)	1	(Ref)	1	(Ref)
1–2 hrs	150 (20.4)	2.43	(0.89;6.66)	1.86	(0.92;3.75)
2–3 hrs	140 (19.0)	2.86	(0.98;8.34)	1.86	(0.90;3.85)
More than 3 hrs	287 (38.9)	3.02	(1.17;7.77)	2.84	(1.51;5.35)
P-value (test for trend)		0.029		0.001	
Call reciprocity*	* *	* *	* *	* *	* *
0–3 reciprocated ties	145 (19.9)	1	(Ref)	1	(Ref)
4–6 reciprocated ties	245 (33.7)	1.31	(0.50;3.39)	1.44	(0.72;2.88)
7–9 reciprocated ties	167 (22.9)	2.60	(0.98;6.90)	2.61	(1.30;5.26)
More than 9	171 (23.5)	2.04	(0.73;5.74)	3.47	(1.74;6.93)
P-value (test for trend)		0.069		<0.0001	
*Text reciprocity*	* *	* *	* *	* *	* *
0–6 reciprocated ties	82 (11.1)	1	(Ref)	1	(Ref)
7–12 reciprocated ties	205 (27.8)	2.23	(0.61;8.12)	1.72	(0.75;3.92)
13–18 reciprocated ties	209 (28.4)	1.89	(0.50;7.13)	1.56	(0.67;3.59)
More than 18	241 (32.7)	4.09	(1.09;15.31)	2.23	(0.98;5.08)
P-value (test for trend)		0.040		0.079	

OR = Odds ratio, 95%CI = 95% confidence interval. All OR adjusted for age, gender, and co-habitation.

*9 observations excluded because of no calling activity.

## Discussion

In this study following more than 700 young adults with survey data and continuous smartphone recordings, we attempted to investigate whether smartphone measures of interactions would be associated with specific survey measures based on the assumption that they to some extend would measure the same underlying theoretical concepts. Confirming this hypothesis, we found considerable associations between self-reports and smartphone measures of social relations. Being in contact with a high number of different individuals via both calls and text messages was associated with reporting to have frequent contact with a high number of social roles. Further, having a high total frequency of calls and text messages, high duration of calls, and high call and text reciprocity was also associated with self-reporting a high contact frequency.

### Social integration

To the best of our knowledge, this is the first study to investigate associations between self-reported social role diversity and social network size in phone communication. Miritello et al. (2013) investigated social network size in individual networks among 20 million mobile users [29]. They did not compare this measure against self-reports but detected similar social connectivity patterns often found in self-reported social networks, where individuals have an upper limit for the number of alters included in the network. One study investigating diversity in communication behavior found that poor mental health was related to smaller communication networks [8].

Even though the current study shows promising results for measuring social integration aspects with smartphone data, one should be aware that the used self-report measure did not evaluate all aspects of social integration. Contrary to other survey instruments evaluating social role diversity [27], the used survey instruments did not measure roles relating to leisure or local community activities, and hence we were not able to consider this aspect of social integration. One study found associations between self-reported phone use and spending leisure time with others with the underlying hypothesis that individuals keep in contact via mobile phones before meeting and organizing events [42]. However, further research is needed to clarify whether objectively measured smartphone communication is associated with leisure and local community activities. Other dimensions of social integration which have rarely been investigated for a larger population due to data limitations of survey self-reports are concepts such as density in social networks, and centrality in social networks [43]. These measures are difficult to obtain on a large scale with self-reports as this requires extensive mapping of whole (i.e. socio-centric) networks. Smartphone data provide possibilities to also investigate social networks beyond the individual level if data is collected with a considerable high coverage within a somewhat specifically defined environment, e.g. neighborhood, workplace, or educational institution. In the study we were not able to consider such measures because of the relatively low response rate.

The differences in the two measures used to operationalize social integration, i.e. social role diversity and social network size should also be noted. The survey items used were only able to evaluate contact with up to six different social roles, whereas there might be considerable more diversity in a network of communication. Further, the number of alters included in the social role “friend” vary from person to person meaning that the diversity in phone contacts might differ more than what can be captured with the survey item of social role diversity used. This point might also be especially relevant considering the population under study consisting of college students who possibly have a high contact frequency with a range of friends. Social roles can be viewed as sets of behavioral contingencies which are a result of the social interactions in one’s proximal environment [27]. Because the social environment of young adulthood in college is likely primarily comprised of a milieu of peers, the behavioural expectations and cues that emerge from learned behaviours in this social environment are largely based on expectancies formed from peer-to-peer interactions. As such, it is likely that college students maintain fewer social roles in comparison to adults during this transitional developmental time period of college as they spend most of their time interacting socially with peers rather than engaging in many diverse social roles. Hence, it is possible that smartphone measures are actually a more valid representation of the construct of social integration for a population of young adults given that they are in college and they interact with peers via smartphone devices. Challenges using the smartphone for estimating social network size, nevertheless, also include a risk of overestimation due to service calls and alike. We tried to minimize this measurement error by conducting sensitivity analyses restricting social contacts called at least three times.

### Tie strength

We also found considerable associations between self-reported and smartphone recorded contact frequency. Contact frequency via smartphones has been found in other studies to predict tie strength. In a study by Saramäki et al., they found that the frequency of calls to a specific alter predict self-reported closeness as well as self-reported number of days since last face-to- face encounter with the same individual [34]. Another study found that self-reported emotional closeness was related to having short time between phone communication events [44]. Hence, there appears to build-up evidence that contact frequency via phone communication may be a good predictor of tie strength. Predicting self-reports of tie strength with reciprocity in and duration of smartphone communication has rarely been done, but we found convincing evidence for an association between the two data sources with respect to both reciprocity and duration. In the study by Miritello et al. distributions of tie strength as duration of phone calls was investigated [29]. They did not compare this measure against self-reports, but they detected constraints on the number of strong ties in large networks suggesting similar social connectivity patterns often found in self-reported social networks, where individuals have an upper limit for strong ties. Although frequency, duration and reciprocity in call and text patterns might be good proxies for functional dimensions of tie strength such as closeness and intimacy these functional dimensions might be complex to study directly with smartphone data as evaluation of such aspects would require access to the content of the calls and text messages.

### Privacy and data access

Along with the increasing usage of smartphone data, privacy issues is a concern especially when merged with sensitive data concerning health and well-being [45]. On the other hand, the scientific community may also have obligations to take on and explore new possibilities in data and technology that can potentially contribute to the improvement of the public health of populations [46, 47]. Whereas collection of smartphone data requires very little efforts of the study participants, it still remains a challenge to access the digital traces from smartphones. Most national phone companies hold large scale smartphone generated data that could constitute basis for research. Nevertheless, companies can be reluctant to share these data due to privacy issues or own commercial interests, and often the research community does not have direct access to large scale smartphone data [48]. Digital traces from social media also constitute valuable information on social relations. Nevertheless, one should keep in mind that the representativeness of social media data might be compromised by user preferences for the specific social media [49, 50], whereas this might be less of a problem with smartphone data such as calls and text messages as the coverage in most of the western world is high [40]. Collecting smartphone data a priori with informed consent as done in the present study allows direct access to relevant smartphone interactions as well as merging with other survey and health administration data, but this approach is feasible on a smaller scale.

### Strengths and limitations

This study used data from one of the largest databases containing detailed information on both continuous smartphone recordings around the clock linked to relevant self-reports from surveys. Nevertheless, the response rate of 29% should be taken into consideration when interpreting the results. Unfortunately, we did not have data available to further explore characteristics of non-responders. Further, one should be aware that the study population does not constitute a random sample of the population but reflect a selected population of young adults attending higher education, who were likely to interact socially. The pattern of and motivations for smartphone use differ by age [39], and hence the results might be less generalizable to older age groups.

### Conclusion

The use of digital traces from social interactions in the health and social science research is inevitable. We have shown a considerable overlap between self-reported and smartphone derived measures of social relations and conclude that smartphone data hold promising potential for a detailed measurement of social interaction and social relations, which can be used as supplementary information to established survey measures.

## Supporting information

S1 Table Associations between age, gender and smartphone measures of social relations in a population of 737 young adults. (DOCX)

S2 Table Associations between age, gender and self-reported measures of social relations in a population of 737 young adults. (DOCX)

S3 Table Linear regression of associations between continuous smartphone measures and self-reported social relation variables. (DOCX)

S4 Table Associations between smartphone and self-reported measures of social integration in a population of 737 young adults excluding co-habiting individuals. (DOCX)

S5 Table Associations between self-reported and smartphone measures of social integration in a population of 737 young adults. Restricted social network size to a minimum of three social interactions per unique alter.
(DOCX)
