# Supplementary material for: Measuring social integration and tie strength with smartphone and survey data
Source: PLoS One. 2018 Aug 23;13(8):e0200678. doi: 10.1371/journal.pone.0200678 (PMC6107109; doi:10.1371/journal.pone.0200678)
Supplement: S1 Table — (DOCX) [file pone.0200678.s001.docx]

| **S1 Table: Associations between age, gender and smartphone measures of social relations in a population of 737 young adults** | | | | |
| --- | --- | --- | --- | --- |
|  | **Total population** | **Gender** | | **Age** |
|  | **N (col %)** | **Men N (col%)** | **Women N (col%)** | **Mean (SD)** |
| **Number of alters called** |  |  |  |  |
| 0-10 alters | 186 (25.2) | 151 (26.6) | 35 (20.7) | 21.1 (2.5) |
| 11-20 alters | 286 (38.8) | 218 (38.4) | 68 (40.2) | 21.7 (2.8) |
| 21-30 alters | 181 (24.6) | 135 (23.8) | 46 (27.2) | 21.7 (2.7) |
| More than 30 alters | 84 (11.4) | 64 (11.3) | 20 (11.8) | 22.1 (2.1) |
| P-value |  | 0.46 | | 0.019 |
| **Number of alters texted** |  |  |  |  |
| 0-10 alters | 92 (12.5) | 79 (13.9) | 13 (7.7) | 21.1 (3.0) |
| 11-20 alters | 298 (40.4) | 248 (43.7) | 50 (29.6) | 21.7 (2.9) |
| 21-30 alters | 223 (30.3) | 162 (28.5) | 61 (36.1) | 21.5 (2.5) |
| More than 30 alters | 124 (16.8) | 79 (13.9) | 45 (26.6) | 21.8 (1.9) |
| P-value |  | <0.0001 | | 0.29 |
| **Frequency of call interactions per alter** |  |  |  |  |
| 0-3 calls | 221 (30.0) | 179 (31.5) | 42 (24.9) | 21.5 (2.4) |
| 4-6 calls | 380 (51.6) | 290 (51.1) | 90 (53.3) | 21.7 (2.8) |
| 7-9 calls | 96 (13.0) | 69 (12.1) | 27 (16.0) | 21.7 (2.7) |
| More than 9 calls | 40 (5.4) | 30 (5.3) | 10 (5.9) | 20.9 (2.0) |
| P-value |  | 0.31 | | 0.29 |
| **Frequency of text interactions per alter** |  |  |  |  |
| 0-14 texts | 298 (40.4) | 255 (44.9) | 43 (25.4) | 22.0 (3.2) |
| 15-29 texts | 240 (32.6) | 182 (32.0) | 58 (34.3) | 21.7 (2.1) |
| 30-45 texts | 100 (13.6) | 61 (10.7) | 39 (23.1) | 21.1 (2.0) |
| 45 or more texts | 99 (13.4) | 70 (12.3) | 29 (17.2) | 20.8 (2.0) |
| P-value |  | <0.0001 | | 0.0003 |
| **Call duration** |  |  |  |  |
| up to 1 hr | 160 (21.7) | 141 (24.8) | 19 (11.2) | 21.0 (2.2) |
| 1-2 hrs | 150 (20.4) | 120 (21.1) | 30 (17.8) | 21.4 (2.8) |
| 2-3 hrs | 140 (19.0) | 105 (18.5) | 35 (20.7) | 21.7 (2.5) |
| More than 3 hrs | 287 (38.9) | 202 (35.6) | 85 (50.3) | 22.0 (2.8) |
| P-value |  | 0.0002 | | 0.001 |
| ***Call reciprocity**** |  |  |  |  |
| 0-3 reciprocated ties | 145 (19.9) | 116 (20.7) | 29 (17.3) | 21.4 (2.6) |
| 4-6 reciprocated ties | 245 (33.7) | 187 (33.4) | 58 (34.5) | 21.4 (2.4) |
| 7-9 reciprocated ties | 167 (22.9) | 123 (22.0) | 44 (26.2) | 21.9 (3.4) |
| More than 9 | 171 (23.5) | 134 (23.9) | 37 (22.0) | 21.7 (2.1) |
| P-value |  | 0.57 | | 0.119 |
| **Text reciprocity** |  |  |  |  |
| 0-6 reciprocated ties | 82 (11.1) | 72 (12.7) | 10 (5.9) | 21.3 (3.1) |
| 7-12 reciprocated ties | 205 (27.8) | 168 (29.6) | 37 (21.9) | 21.6 (3.1) |
| 13-18 reciprocated ties | 209 (28.4) | 172 (30.3) | 37 (21.9) | 21.7 (2.8) |
| More than 18 | 241 (32.7) | 156 (27.5) | 85 (50.3) | 21.6 (1.8) |
| P-value |  | <0.0001 | | 0.65 |
| **Total** | 737 (100) | 568 (100) | 169 (100) | 21.6 (2.6) |
| *Nine individuals did not have call activity and were excluded from this variable. | | |  |  |
