# Supplementary material for: Measuring social integration and tie strength with smartphone and survey data
Source: PLoS One. 2018 Aug 23;13(8):e0200678. doi: 10.1371/journal.pone.0200678 (PMC6107109; doi:10.1371/journal.pone.0200678)
Supplement: S2 Table — (DOCX) [file pone.0200678.s002.docx]

| **S2 Table: Associations between age, gender and self-reported measures of social relations in a population of 737 young adults** | | | | |
| --- | --- | --- | --- | --- |
|  | **Total population** | **Gender** | | **Age** |
|  | **N (col %)** | **Male N (col%)** | **Female N (col%)** | **Mean (SD)** |
| **Number of roles in frequent contact with (face-to-face)** |  |  |  |  |
| 0-2 roles | 150 (20.4) | 119 (21.0) | 31 (18.3) | 22.2 (3.0) |
| 3-4 roles | 280 (38.0) | 217 (38.2) | 63 (37.3) | 21.7 (3.0) |
| 5-6 roles | 307 (41.7) | 232 (40.8) | 75 (44.4) | 21.2 (2.0) |
| *P-value* |  | 0.65 | | 0.0002 |
| **Number of roles in frequent contact with (non-face-to-face)** |  |  |  |  |
| 0-2 roles | 28 (3.8) | 25 (4.4) | 3 (1.8) | 22.4 (4.7) |
| 3-4 roles | 290 (39.3) | 229 (40.3) | 61 (36.1) | 21.6 (2.6) |
| 5-6 roles | 419 (56.9) | 314 (55.3) | 105 (62.1) | 21.5 (2.5) |
| *P-value* |  | 0.13 | | 0.27 |
| **Total contact frequency with all roles (summary score)** |  |  |  |  |
| 0-9 | 113 (15.3) | 88 (15.5) | 25 (14.8) | 22.3 (3.7) |
| 10-14 | 324 (44.0) | 255 (44.9) | 69 (40.8) | 21.9 (2.7) |
| 15-19 | 255 (34.6) | 188 (33.1) | 67 (39.6) | 21.1 (2.0) |
| 20-24 | 45 (6.1) | 37 (6.5) | 8 (4.7) | 20.4 (1.0) |
| *P-value* |  | 0.42 | | <0.0001 |
| **Total non-face-to-face contact frequency with all roles (summary score)** | |  |  |  |
| 0-9 | 40 (5.4) | 37 (6.5) | 3 (1.8) | 21.9 (4.0) |
| 10-14 | 262 (35.6) | 209 (36.9) | 53 (31.4) | 21.8 (2.8) |
| 15-19 | 320 (43.5) | 241 (42.5) | 79 (46.7) | 21.5 (2.5) |
| 20-24 | 114 (15.5) | 80 (14.1) | 34 (20.1) | 21.1 (2.1) |
| *Missing* | 1 |  |  |  |
| *P-value* |  | 0.018 | | 0.10 |
| **Co-habiting (Living with at least one of six roles)** |  |  |  |  |
| No | 422 (57.3) | 326 (57.4) | 96 (56.8) | 21.5 (3.1) |
| Yes | 315 (42.7) | 242 (42.6) | 73 (43.2) | 21.7 (2.2) |
| *P-value* |  | 0.96 | | 0.42 |
| **Number of no roles (Reporting 'Have no')** |  |  |  |  |
| No missing roles | 310 (42.1) | 232 (40.8) | 78 (46.2) | 21.9 (2.8) |
| One missing role | 391 (53.1) | 306 (53.9) | 85 (50.3) | 21.3 (2.0) |
| Two missing roles | 34 (4.6) | 29 (5.1) | 5 (3.0) | 22.5 (5.9) |
| Three missing roles | 2 (0.3) | 1 (0.2) | 1 (0.6) | 21.5 (3.5) |
| *P-value* |  | 0.35 | | 0.003 |
