# Supplementary material for: Measuring social integration and tie strength with smartphone and survey data
Source: PLoS One. 2018 Aug 23;13(8):e0200678. doi: 10.1371/journal.pone.0200678 (PMC6107109; doi:10.1371/journal.pone.0200678)
Supplement: S3 Table — (DOCX) [file pone.0200678.s003.docx]

| **S3A Table: Linear regression of associations between continuous self-reported and smartphone measures of social integration** | | | | | | |
| --- | --- | --- | --- | --- | --- | --- |
|  | **Social Integration** | | | | | |
|  | **Self-reported social role diversity (Face-to-face contact)** | | | **Self-reported social role diversity (Non-face-to-face contact)** | | |
| **Smartphone measures** | **Coef.** | **95%CI** | **p-value** | **Coef.** | **95%CI** | **p-value** |
| Social network size (calls) | 0.01 | [-0.00;0.02] | 0.071 | 0.02 | [0.01;0.03] | <0.001 |
| Social network size (texts) | 0.01 | [0.00;0.02] | 0.016 | 0.02 | [0.01;0.02] | <0.001 |
| 95%CI= 95% confidence interval. All OR adjusted for age, gender and co-habitation. | | | | | | |

| **S3B Table: Linear regression of associations between continuous self-reported and smartphone measures of tie strength** | | | | | | |
| --- | --- | --- | --- | --- | --- | --- |
|  | **Tie strength** | | | | | |
|  | **Total face-to-face contact frequency** | | | **Total non-face-to-face contact frequency** | | |
| **Smartphone measures** | **Coef.** | **95%CI** | **p-value** | **Coef.** | **95%CI** | **p-value** |
| Frequency of call interactions | 0.17 | [0.08;0.26] | <0.001 | 0.29 | [0.19;0.39] | <0.001 |
| Frequency of text interactions | 0.01 | [0.01;0.02] | 0.001 | 0.02 | [0.01;0.03] | <0.001 |
| Call duration | 0.00 | [-0.00;0.0.01] | 0.20 | 0.00 | [0.00;0.01] | < 0.001 |
| Call reciprocity | 0.11 | [0.05;0.17] | <0.001 | 0.20 | [0.14;0.27] | < 0.001 |
| Text reciprocity | 0.05 | [0.02;0.08] | <0.001 | 0.06 | [0.03;10] | < 0.001 |
| 95%CI= 95% confidence interval. All OR adjusted for age, gender and co-habitation. | | | | | | |
