# Supplementary material for: Measuring social integration and tie strength with smartphone and survey data
Source: PLoS One. 2018 Aug 23;13(8):e0200678. doi: 10.1371/journal.pone.0200678 (PMC6107109; doi:10.1371/journal.pone.0200678)
Supplement: S4 Table — (DOCX) [file pone.0200678.s004.docx]

| **S4 Table: Associations between smartphone and self-reported measures of social integration in a population of 737 young adults excluding 315 co-habiting individuals** | | | |
| --- | --- | --- | --- |
|  | **Total population** | **High self-reported social role diversity (Frequent face-to-face contact with 5-6 social roles)** | |
| **Smartphone social netwok size per month** | **N (%)** | **OR** | **95%CI** |
| *Number of alters called* |  |  |  |
| 0-10 alters | 95 (22.5) | 1 | [Ref] |
| 11-20 alters | 150 (35.5) | 1.41 | [0.79;2.52] |
| 21-30 alters | 117 (27.7) | 1.85 | [1.02;3.36] |
| More than 30 alters | 60 (14.2) | 1.92 | [0.95;3.88] |
| P-value (test for trend) |  | 0.032 | |
| *Number of alters texted* |  |  |  |
| 0-10 alters | 43 (10.2) | 1 | [Ref] |
| 11-20 alters | 149 (35.3) | 1.79 | [0.79;4.06] |
| 21-30 alters | 141 (33.4) | 2.48 | [1.09;5.65] |
| More than 30 alters | 89 (21.1) | 2.09 | [0.87;5.03] |
| P-value (test for trend) |  | 0.092 | |
| OR= Odds ratio, 95%CI= 95% confidence interval. All OR adjusted for age and gender. | | | |
