# Supplementary material for: Measuring social integration and tie strength with smartphone and survey data
Source: PLoS One. 2018 Aug 23;13(8):e0200678. doi: 10.1371/journal.pone.0200678 (PMC6107109; doi:10.1371/journal.pone.0200678)
Supplement: S5 Table — Restricted social network size to a minimum of three social interactions per unique alter. (DOCX) [file pone.0200678.s005.docx]

| **S5 Table: Sensitivity analysis of associations between self-reported and smartphone measures of social integration in a population of 737 young adults. Restricted social network size to a minimum of three social interactions per unique alter** | | | | | |
| --- | --- | --- | --- | --- | --- |
|  | **Total population** | **High self-reported social role diversity (Frequent face-to-face contact with 5-6 social roles)** | | **High self-reported social role diversity (Frequent non face-to-face contact with 5-6 social roles)** | |
| **Smartphone social network size per month** | **N (%)** | **OR** | **(95%CI)** | **OR** | **(95%CI)** |
| *Number of alters called* |  |  |  |  |  |
| 0-5 alters | 130 (17.6) | 1 | (Ref) | 1 | (Ref) |
| 16-20 alters | 198 (26.9) | 1.00 | (0.63;1.61) | 1.64 | (0.75;3.58) |
| 6-16 alters | 170 (23.1) | 1.51 | (0.93;2.45) | 3.56 | (1.69;7.49) |
| More than 20 alters | 239 (32.4) | 1.57 | (0.99;2.48) | 4.84 | (2.36;9.92) |
| P-value (test for trend) |  | 0.011 | | <0.0001 | |
| *Number of alters texted* |  |  |  |  |  |
| 0-6 alters | 87 (11.8) | 1 | (Ref) | 1 | (Ref) |
| 7-12 alters | 230 (31.2) | 2.04 | (1.17;3.55) | 2.54 | (1.09;5.93) |
| 13-18 alters | 205 (27.8) | 2.39 | (1.36;4.21) | 2.51 | (1.06;5.94) |
| More than 18 alters | 215 (29.2) | 2.56 | (1.44;4.54) | 4.15 | (1.77;9.76) |
| P-value (test for trend) |  | 0.004 | | 0.001 | |
| OR= Odds ratio, 95%CI= 95% confidence interval. All OR adjusted for age, gender and co-habitation. | | | | | |
